# How cholesterol interacts with membrane proteins: an exploration of cholesterol-binding sites including CRAC, CARC, and tilted domains

**DOI:** 10.3389/fphys.2013.00031

**Published:** 2013-02-28

**Authors:** Jacques Fantini, Francisco J. Barrantes

**Affiliations:** ^1^EA-4674, Interactions Moléculaires et Systèmes Membranaires, Aix-Marseille UniversitéMarseille, France; ^2^Laboratory of Molecular Neurobiology, Faculty of Medical Sciences, Biomedical Research Institute (BIOMED) UCA–CONICET, Catholic University of ArgentinaBuenos Aires, Argentina

**Keywords:** cholesterol, CH-Pi, lipid-protein interaction, lipid raft, neurotransmitter, receptor structure, alpha-synuclein, Alzheimer

## Abstract

The plasma membrane of eukaryotic cells contains several types of lipids displaying high biochemical variability in both their apolar moiety (e.g., the acyl chain of glycerolipids) and their polar head (e.g., the sugar structure of glycosphingolipids). Among these lipids, cholesterol is unique because its biochemical variability is almost exclusively restricted to the oxidation of its polar −OH group. Although generally considered the most rigid membrane lipid, cholesterol can adopt a broad range of conformations due to the flexibility of its isooctyl chain linked to the polycyclic sterane backbone. Moreover, cholesterol is an asymmetric molecule displaying a planar α face and a rough β face. Overall, these structural features open up a number of possible interactions between cholesterol and membrane lipids and proteins, consistent with the prominent regulatory functions that this unique lipid exerts on membrane components. The aim of this review is to describe how cholesterol interacts with membrane lipids and proteins at the molecular/atomic scale, with special emphasis on transmembrane domains of proteins containing either the consensus cholesterol-binding motifs CRAC and CARC or a tilted peptide. Despite their broad structural diversity, all these domains bind cholesterol through common molecular mechanisms, leading to the identification of a subset of amino acid residues that are overrepresented in both linear and three-dimensional membrane cholesterol-binding sites.

## Introduction

Transmembrane domains of proteins cross the lipid bilayer of biological membranes to ensure the insertion of a subset of amino acid residues within the membrane. These proteins are usually referred to as integral membrane proteins. Deciphering the molecular mechanisms allowing the interaction of these proteins with membrane lipids has been and still is the subject of intense research efforts at the crossroad of several scientific areas including biochemistry, biophysics, cell biology, and bioinformatics. Basically, a transmembrane domain is an α-helical segment of ca. 20–25 apolar amino acid residues flanked at each end by more polar residues allowing them to “float” at the lipid-water interface region of the membrane, thereby stabilizing the helix within the lipid bilayer (Lee, 2003). These “interfacial” amino acid residues have to manage the transition between an apolar and a polar environment. For this reason, Lys, Arg, Tyr, and Trp residues are most commonly found at these flanking positions. Lys and Arg have a long apolar side chain buried in the apolar section of the membrane, and a positively charged basic group that “breathes” at the surface of the membrane. This unique topology and its associated functional counterpart are metaphorically referred to as the “snorkeling” effect (Strandberg and Killian, 2003). By the same token, Trp, and Tyr have an aromatic structure compatible with the apolar region of the membrane, but contain an −OH group (Tyr) or an N atom (Trp) capable of forming hydrogen bonds with polar groups (Lee, 2003). Nonhelical transmembrane β structures have also been characterized, for instance in bacterial cytolysins (Harris and Palmer, 2010), but are less represented than the widespread α-helical domains in resident plasma membrane proteins. In this review, we will focus on TM domains with an α-helical structure and their different modes of interaction with membrane cholesterol.

## Cholesterol structure, dynamics, and membrane topology

**Figure 1:**
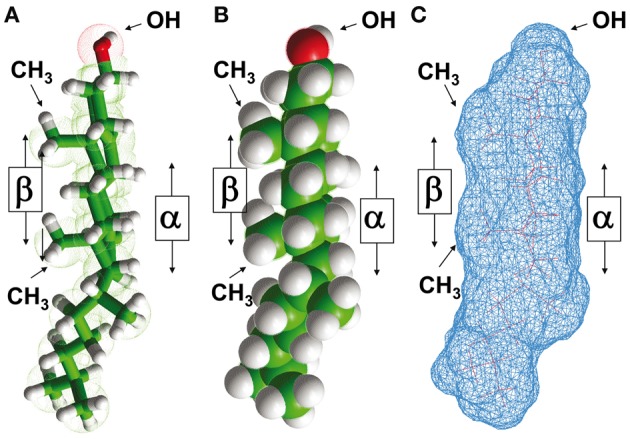
**Structural properties of cholesterol.** The asymmetric distribution of aliphatic groups (methyl, iso-octyl) linked to the planar sterane backbone of cholesterol defines two distinct sides referred to as α and β faces, according to the nomenclature of ring compounds proposed by Rose et al. (1980). This asymmetric structure of cholesterol is illustrated in a tube model **(A)**, a sphere model **(B)**, and a molecular surface model **(C)**. Note that the OH group is closer to the “smooth” α face than to the “rough” β face.

Cholesterol is a polycyclic amphipathic molecule derived from the sterane backbone (Fantini and Barrantes, 2009). Its polar section is restricted to a single hydroxyl (OH) group which can form two distinct types of hydrogen bond (acceptor and donor) with a polar group belonging to either a membrane lipid or a protein. The apolar section of cholesterol has an asymmetric structure with two distinct faces, referred to as α and β according to the system numeration of ring compounds proposed by Rose et al. (1980) (Figures 1A and B). The α face displays a planar surface, in contrast with the β face which has a significantly rougher surface owing to the presence of several aliphatic groups (two methyl groups and a terminal isooctyl chain that are linked to the sterane backbone) (Fantini and Barrantes, 2009) (Figure 1C). The side chains of branched amino acids such as Ile, Val, or Leu can interpenetrate these aliphatic “spikes” and are thus particularly suited for an association with the β face of cholesterol through van der Waals interactions. This is the case for the cholesterol binding domain of α-synuclein (Fantini et al., 2011). Moreover, aromatic side chains can stack onto the α face of cholesterol through CH-π interactions (Nishio et al., 1995). However, this should not be taken as an absolute rule since the aliphatic side chains of an α-helical segment could also form a groove with a planar surface fitting the α face of cholesterol (Di Scala et al., 2013). Conversely, an aromatic ring oriented normally with respect to the main axis of an α-helical region could perfectly well accommodate the rough β face of cholesterol by intercalating the aromatic structure between the aliphatic spikes of the lipid.

**Figure 2:**
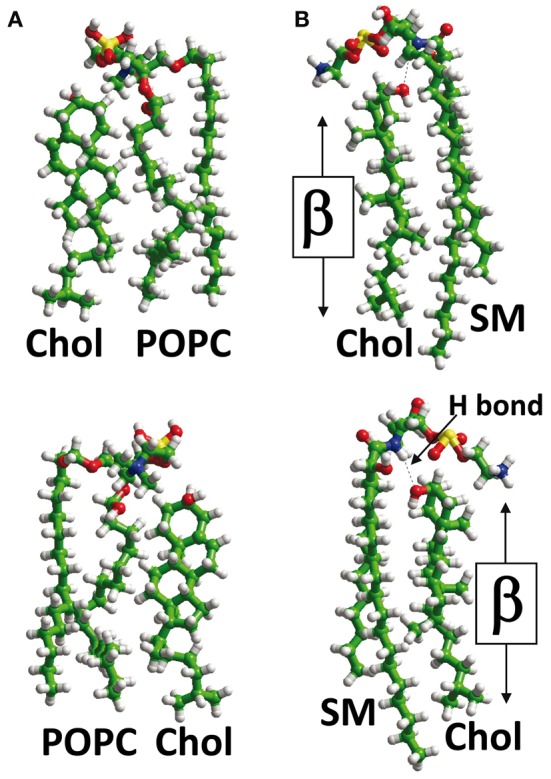
**Lipid-cholesterol interactions.** In the plasma membrane, cholesterol (Chol) can interact with phosphatidylcholine, e.g., palmitoyl-oleyl-phosphatidylcholine (POPC) (panel **A**) or sphingolipids such as sphingomyelin (panel **B**). When cholesterol interacts with POPC, its OH group is not buried in the complex, and both its α and β faces are available for TM domains of proteins **(A)**. However, when cholesterol interacts with SM, a hydrogen bond (H bond) is formed between the OH group of cholesterol and the NH group of the sphingolipid. This H bond orientates cholesterol with respect to SM so that only its β face remains available for TM domains. The OH group of cholesterol is masked by the polar head of sphingomyelin in a typical “umbrella” effect.

**Figure 3:**
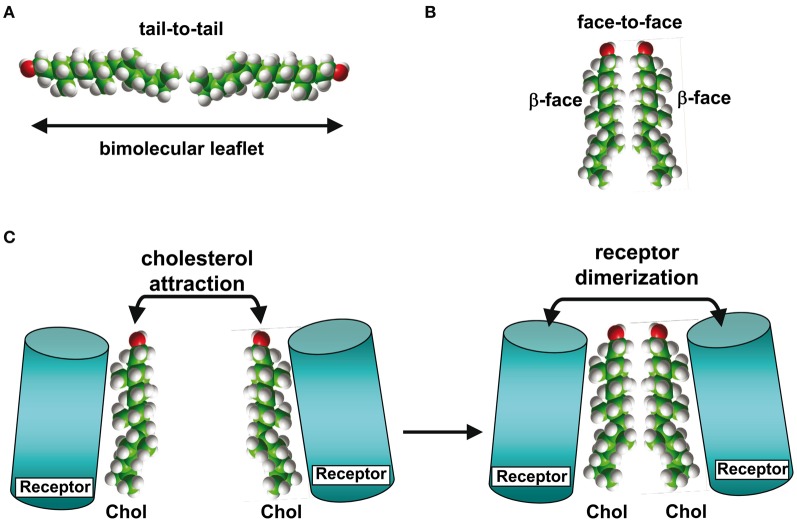
**Cholesterol-cholesterol interactions.** In model membranes, two cholesterol molecules can form a tail-to-tail **(A)** or a face-to-face **(B)** complex. In the latter case, the self-recognition properties of cholesterol can induce the dimerization of membrane receptors **(C)**, as demonstrated for G-protein-coupled receptors with 7-TM domains.

Another key parameter which determines how cholesterol interacts with a TM domain of a protein is the membrane phase to which it belongs. It should be kept in mind that although cholesterol is concentrated in sphingolipid-enriched membrane microdomains such as lipid “rafts” (Simons and Ikonen, 1997; Anderson and Jacobson, 2002) it is also present outside these microdomains, i.e., in the liquid disordered (Ld) phase of the plasma membrane that contains high amounts of glycerophospholipids such as phosphatidylcholine (Fantini et al., 2002). Studies with model membranes indicate that cholesterol interacts more favorably with sphingomyelin than with phosphatidylcholine (Mattjus and Slotte, 1996) (Figure 2), a behavior which has been attributed to the presence of a saturated acyl chain in sphingomyelin compared to a cis-unsaturated chain in phosphatidylcholine (Fantini et al., 2002). The saturated chain in sphingomyelin, together with the trans-unsaturated sphingosine backbone, would allow maximal van der Waals interactions with cholesterol. Moreover, phosphatidylcholine (Figure 2A) has carbonyl groups which could act as hydrogen bond acceptors, but is devoid of any hydrogen bond donor group such as the amino group of sphingomyelin (Figure 2B). Therefore, the association between cholesterol and phosphatidylcholine relies on weakly discriminative van der Waals forces plus limited hydrogen bond capabilities. When cholesterol is associated with phosphatidylcholine, both its α and β faces (Rose et al., 1980) are potentially available for an interaction with a TM domain (Figure 2A). Moreover, the −OH group of cholesterol is not buried in the phosphatidylcholine/cholesterol complex and thus remains accessible for establishing a hydrogen bond with a TM domain. In contrast, cholesterol forms condensed lipid complexes with sphingolipids (either sphingomyelin or glycosphingolipids) as shown in Figure 2B (Radhakrishnan et al., 2000). In these molecular assemblies, the −OH group of cholesterol is available for the formation of a stabilizing hydrogen bond with the polar head group of the sphingolipid. Thus, this −OH group is not initially available for a hydrogen bond with a TM domain of a protein. Sphingolipids generally interact with the α face of cholesterol, leaving the β face available for the TM domain (Fantini and Barrantes, 2009). Finally, it is interesting to note that in model membranes, cholesterol can form two distinct types of dimers that are stabilized through van der Waals interactions: (1) transbilayer tail-to-tail dimers (Harris et al., 1995; Mukherjee and Chattopadhyay, 1996; Rukmini et al., 2001) (Figure 3A); and (2) dimers formed by the association of two cholesterol molecules interacting with their respective smooth α faces, leaving the opposite β faces available for protein binding (Figure 3B). In this latter case, the cholesterol dimer can recruit for instance two G-protein coupled receptors, thereby inducing their functional dimerization (Figure 3C) (Hanson et al., 2008). That such cholesterol dimers actually exist in natural plasma membranes has not been formally demonstrated. Although the intracellular dynamics of cholesterol has been the subject of intense research efforts during the last decade (Mesmin and Maxfield, 2009), the transbilayer distribution of cholesterol has remained uncertain (Ikonen, 2008). Indeed, despite the fact that cholesterol preferentially interacts with sphingolipids in the exofacial leaflet, fluorescence quenching studies suggested that it is in fact more abundant in the cytoplasmic leaflet (Mondal et al., 2009). The molecular basis for such an asymmetric transbilayer distribution of plasma membrane cholesterol is unknown. However, the limited accessibility of cholesterol in the exofacial leaflet, due to its tight interactions with sphingolipids, could lead to an underestimation of its content in this leaflet. Nevertheless, whichever membrane layer has the highest cholesterol content, cholesterol is present in both leaflets of the plasma membrane, providing various interaction possibilities with TM domains. In all cases, unraveling the molecular mechanisms involved in the interaction of TM domains with cholesterol may require (1) the description of the cholesterol/α-helix complex at the molecular/atomic level and (2) consideration of the lipid/protein environment in which this interaction occurs. With this in mind we will now describe the different types of protein structures capable of interacting with membrane cholesterol.

## The CRAC domain

There is little doubt that the most popular cholesterol-binding domain in the scientific literature is the **C**holesterol **R**ecognition/interaction **A**mino acid **C**onsensus sequence, generally referred as the **CRAC** domain (Li and Papadopoulos, 1998). CRAC is a short linear motif which fulfills a very simple algorithm, that is, in the N-terminus to C-terminus direction: a branched apolar Leu or Val residue, followed by a segment containing 1–5 of any residues, then an aromatic residue that is mandatory Y, then again a segment containing 1–5 of any residues, and finally a basic Lys or Arg, i.e., in the one letter amino acid code, (L/V)-X_1−5_-(Y)-X_1−5_-(K/R). The looseness of the CRAC definition for a motif that mediates binding to a unique lipid that does not display any structural variation is rather unexpected, raising some skepticism about its predictive value (Palmer, 2004; Epand, 2006). However, the motif has been found in various proteins known to bind cholesterol and in many cases the interaction between cholesterol and CRAC has been confirmed by physicochemical approaches. Moreover, single mutations in the CRAC domain have been found to markedly decrease or abolish the interaction, as is the case for the central aromatic residue which is necessarily Tyr and cannot be replaced by any other aromatic residue (Jamin et al., 2005; Epand, 2006; Epand et al., 2006).

**Figure 4:**
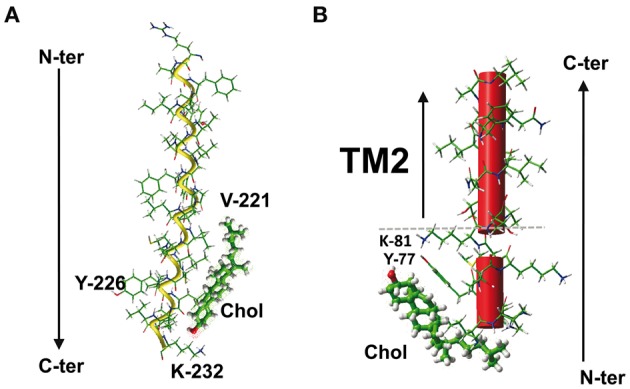
**The CRAC/cholesterol complex in a membrane environment. (A)** Docking of cholesterol on the CRAC domain of the TM5 domain of human type 3 somatostatin receptor. The CRAC domain (221-**V**ICLC**Y**LLIVV**K**K-232) is located in the cytoplasmic leaflet of the membrane bilayer. Note that the central Y-226 residue of CRAC is not involved in cholesterol interaction. The total energy of interaction has been estimated at −43 kJ.mol^−1^ (Baier et al., 2011). **(B)** Docking of cholesterol on the CRAC domain close the TM2 domain of human delta-type opioid receptor. In this case, the CRAC domain (fragment 74-**IV**R**Y**T**K**M**K**-81) lies outside the membrane. The aromatic side chain of Y-77 plays a critical role in this interaction (see Figure 5). The polar-apolar interface of the membrane is indicated by a dotted gray line.

Molecular modeling studies have shown that the CRAC motif belonging to TM domains can have a good fit for cholesterol, as illustrated for the 5th TM domain of the human type 3 somatostatin receptor (Figure 4A). This CRAC domain lies between amino acid residues 221–231 and has the following sequence: VICLCYLLIVVK. It can therefore be either interpreted as **L**C**Y**LLIVV**K** or as **V**ICLC**Y**LLIVV**K** (amino acid residues that fulfill the CRAC algorithm in bold and underlined). Detailed analysis of the energy of interaction between cholesterol and this CRAC domain showed that the complex involves essentially five residues, four of which belong to the CRAC motif (V-221, C-225, L-228, and I-229) and the fifth remaining outside (K-232), for a total energy of interaction of −43 kJ.mol^−1^ (Baier et al., 2011). Surprisingly, the central Tyr residue does not interact with cholesterol, although it is mandatory in the definition of CRAC. Moreover, the energetic pattern of the cholesterol-binding site, i.e., 221-**V**ICL**C**YL**L**IVVK**K**-232 (residues involved in cholesterol binding in bold and underlined) does not overlap with any of the two CRAC motifs defined above (**L**C**Y**LLIVV**K** or **V**ICLC**Y**LLIVV**K**). Overall this may indicate that although the CRAC algorithm has some predictive value for identifying cholesterol-binding motifs in the TM domain, cholesterol can choose a slightly different fit around the CRAC domain to adjust its shape to the three-dimensional structure of the TM domain.

**Figure 5:**
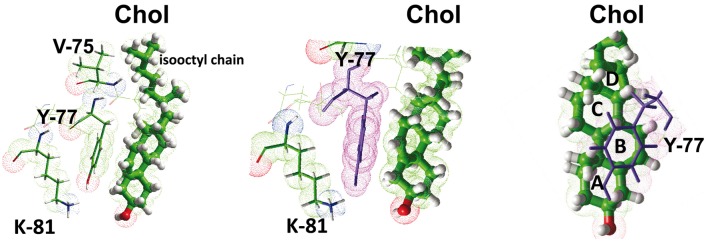
**Molecular mechanisms of cholesterol-CRAC interaction.** This figure shows a detailed analysis of the interaction between cholesterol and the CRAC domain of the human delta-type opioid receptor (see Figure 4). Three distinct views of the complex are shown, with residues I-74, Y-77, and K-81 enlightened. The NH^+^_3_ of K-81, and the OH groups of Y-77 and cholesterol are rejected in a polar area where they can form a network of energetically favored electrostatic interactions (including hydrogen bonds). The aromatic side chain of Y-77 stacks onto the B ring of sterane backbone through typical CH-π stacking interactions. The isooctyl chain of cholesterol interacts with the aliphatic side chains of I-74 (not shown) and V-75. The sterane rings are indicated for cholesterol in the right panel.

There is another caveat in the predictive value of the CRAC algorithm for the specific case of TM domains: the CRAC domain is an oriented motif, with an apolar amino acid residue at the N-ter ending and a highly polar, positively charged basic residue at the C-ter ending. This means that if a CRAC motif belongs to a TM domain and allows this domain to interact with cholesterol, several parameters other than the classical CRAC algorithm have to be fulfilled. First, the basic residue at the C-term ending should be located at the lipid-water interface to ensure an optimal interaction with the membrane structure. Therefore, if the TM domain containing the CRAC motif crosses the membrane in the N-term to C-term direction (i.e., with the N-term region extracellular and the C-term region cytoplasmic), then it will interact with cholesterol in the cytoplasmic leaflet of the plasma membrane (Figure 4A). Conversely, if the TM domain crosses the plasma membrane in the opposite direction (i.e., the N-term region cytoplasmic and the C-term region extracellular), then it will interact with cholesterol in the extracellular leaflet. In both cases, the amino acid residues of the variable segments separating L/V from Y and Y from K/R can still vary, but they must be apolar because they are embedded in the apolar part of the membrane. Since the original definition of the CRAC domain did not specifically take the membrane insertion of the motif into consideration, the X amino acid residues could be any residue. For CRAC motifs belonging to TM domains, the definition should therefore be restricted to (L/V)-X_1−5_-(Y)-X_1−5_-(K/R) with apolar X residues compatible with the hydrophobic membrane environment, otherwise the CRAC algorithm could incorrectly predict the presence of a potential cholesterol-binding domain that in fact lies outside the membrane. This particular case is illustrated for the human delta-type opioid receptor, which contains a CRAC motif the juxtamembrane domain just upstream of the 2nd TM domain (Figure 4B). The sequence of this CRAC domain is 74-I**V**R**Y**T**K**M**K**-81, whereas the 2nd TM domain encompasses residues 85–102. As a matter of fact, the very high polarity of this CRAC motif restricts its location outside the membrane. Nevertheless, as a bona fide CRAC domain, the IVRYTKMK sequence has a high affinity for cholesterol, with a total energy of interaction of −49 kJ.mol^−1^, as calculated from docking studies (Baier et al., 2011). The amino acid residues involved in cholesterol binding are I-74, V-75, R-76, Y-77, and T-78, which can be summarized as a contiguous **IVRYT**KMK motif containing two of the three residues that define the CRAC domain, including the central and mandatory Tyr residue. This aromatic residue (Y-77) contributes −26 kJ.mol^−1^, which represents about 50% of the total energy of interaction of the CRAC/cholesterol complex. Indeed, the side chain of Y-77 binds to cholesterol through a CH-π stacking interaction with the B ring of sterane, whereas its OH group can contribute to a network of electrostatic interactions that also includes the polar OH group of cholesterol (Figure 5). Nevertheless, the predicted membrane topology of the human delta-type opioid receptor suggests that this CRAC domain lies outside the membrane so that its interaction with cholesterol, although *theoretically* possible, is unlikely (Figure 4B). Despite these caveats there are numerous cases of CRAC motifs within TM domains, illustrating the overall robustness of the CRAC algorithm for predicting cholesterol-binding sites in TM segments of integral membrane proteins (Epand, 2006; Epand et al., 2010; Gimpl, 2010; Paila and Chattopadhyay, 2010), including G-protein coupled receptors of several distinct neurotransmitters (Jafurulla et al., 2011; Oddi et al., 2011; Sengupta and Chattopadhyay, 2012). However, in some instances, bioinformatics analysis of the amino acid sequence of some membrane proteins failed to identify any CRAC motifs encased within TM domains. This is the case for the human nicotinic acetylcholine receptor (AChR) and this particular situation led to the development of a new algorithm for predicting cholesterol-binding domains in TM segments of membrane proteins.

## The CARC domain

The new cholesterol-binding domain is similar to the CRAC sequence, but exhibits the opposite orientation along the polypeptide chain (i.e., constitutes an “inverted CRAC” domain), i.e., (K/R)-X_1−5_-(Y/F)-X_1−5_-(L/V) from the N-term ending to the C-term ending (Baier et al., 2011), for which reason the sequence was coined “CARC.” Besides the reverse orientation, CARC is distinct from CRAC in that the central aromatic amino acid can be either Tyr or Phe. Basically, the CARC motif is based on a sequence of amino acids which includes, from N-terminus to C-terminus endings, a basic (Lys or Arg), an aromatic (Tyr or Phe) and a branched aliphatic residue (Leu or Val). The presence of the basic residue ensures that the CARC motif is correctly positioned at the polar-apolar interface of a TM domain, exactly where cholesterol is supposed to be. This is due to the “snorkeling” effect discussed above (Strandberg and Killian, 2003) which can be attributed to the fact that the long and flexible side chain of lysine (or arginine) is buried in the hydrophobic part of the membrane, whereas the cationic group emerges at the membrane surface (Segrest et al., 1990; Baier et al., 2011). Interestingly, Arg and Lys residues are more frequently found in the N-terminal than in the C-terminal section of a TM domain, indicating that snorkeling is an asymmetric phenomenon (Strandberg and Killian, 2003). The specific topology of the CARC sequence (K/R… Y/F… L/V, from the N-terminus to the C-terminus) implies that the key residues following K/R in the motif (i.e., Y/F and L/V) actually belong to a TM domain, and this is consistent with the fact that both aromatic and aliphatic residues have an apolar side chain.

**Figure 6:**
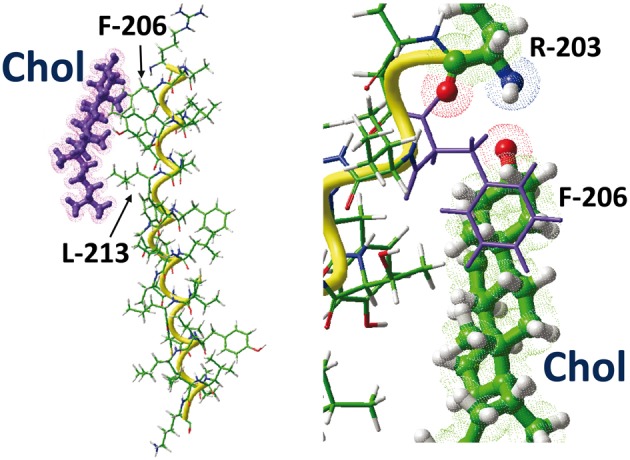
**The CARC/cholesterol complex.** This figure shows the docking of cholesterol on the CARC domains of the TM5 domain of the human type 3 somatostatin receptor. The CARC domain (fragment 203-RAGFIIYTAAL-213) is located in the extracellular leaflet of the TM5 domain of the receptor. Two distinct views of the complex are shown, one with the whole TM5 domain (left panel), the other with residues R-203 and F-206 enlightened. Note the CH-π stacking interaction of the phenyl ring of F-206 onto the A ring of the sterane backbone of cholesterol. The large aliphatic chain of L-213 interacts with the isooctyl group of cholesterol.

Moreover, even if there is no direct interaction between cholesterol and K/R, the basic amino acid in the first position is critical for identifying a functional CARC motif belonging to a TM domain. Incidentally, this also explains why the previously characterized CRAC motif, which has an inverse topology (L/V… Y… K/R, from the N-terminus to the C-terminus), does not always belong to a TM domain (Figure 4B). Furthermore, the tyrosine residue (an absolute requirement for CRAC) can be functionally replaced by Phe in the CARC motif. In the CRAC motif, the phenol group of tyrosine is often required to form a H-bond with the OH group of cholesterol (Epand et al., 2010), and this would not be possible with Phe. In the case of CARC, the interaction between the aromatic amino acid and cholesterol occurs in the apolar region of the membrane, far from the OH group of cholesterol, and the interaction with cholesterol is mediated almost exclusively by the CH-π stacking arrangement between the aromatic ring of the amino acid (either Tyr or Phe) and one of the sterane rings of cholesterol (Figure 6). Finally, the requirement for Leu or Val is justified by the need to accommodate the crevices and asperities of the cholesterol molecule (Fantini and Barrantes, 2009) through numerous van der Waals contacts between these residues and cholesterol. The human type 3 somatostatin receptor illustrates these biochemical principles: in this case, the CARC domain corresponds to the 203-**R**AG**F**II**Y**TAA**L**-213, which overlaps the extracellular leaflet of the 5th domain of the receptor (this TM domain encompasses the segment 206–231). Thus, though as discussed above the Arg residue does not belong to the TM domain, it is important for defining the correct orientation of the TM domain with the CARC motif in the exofacial leaflet of the plasma membrane. All the key amino acid residues that define the CARC motif (R-203, F-206, Y-209, and L-213) do interact with cholesterol, rendering a total energy of interaction of −54 kJ.mol^−1^ (Figure 6). What is particularly interesting in this CARC motif is that it contains not just one but two aromatic residues that both stack onto cholesterol through near-perfect CH-π interactions. Correspondingly, the energetic contribution of these aromatic residues for cholesterol binding is high: −18.2 kJ.mol^−1^ and −14.7 kJ.mol^−1^ for F-206 and Y-209, respectively. The interaction of L-213 with the isooctyl chain of cholesterol accounts for −10.0 kJ.mol^−1^. This illustrates the efficiency of van der Waals interactions between the branched side chain residues and the rough β face of cholesterol. Overall, these unique biochemical features explain why the CARC domains detected in the AChR and its homologous ion channels cover a wide evolutionary span from bacteria to humans (Baier et al., 2011). The CARC motif is also present in the TM domains of the important group of G-protein coupled receptors, which, together with the AChR and other neurotransmitter receptors, are thought to play a role in the pathogenesis of Alzheimer's disease, in connection with high cholesterol content (Barrantes et al., 2010; Thathiah and De Strooper, 2011).

**Figure 7:**
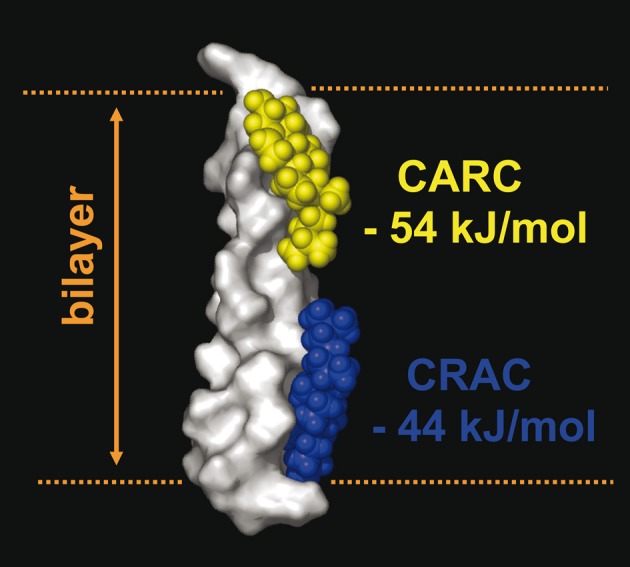
**Occurrence of two cholesterol binding motifs (CRAC and CARC) in the same TM domain.** An example of the simultaneous occurrence of two cholesterol-recognition motifs in the same TM segment is given by the 5th TM domain of the human type 3 somatostatin receptor, which possesses a CRAC domain in the cytoplasmic leaflet (in blue) and a CARC domain in its exofacial leaflet (in yellow). The calculated energy of interaction of each domain with cholesterol is indicated. The surface rendering of the TM domain is particularly suited to visualize the three-dimensional interaction with cholesterol (rendered as space fill models).

Finally, it is worth noting that the same TM domain can contain both a CRAC and a CARC sequence, allowing the simultaneous binding of two cholesterol molecules, one in each membrane leaflet, in a tail-to-tail configuration. This case is illustrated in the 5th TM domain of the human type 3 somatostatin receptor (CRAC in the cytoplasmic leaflet, and CARC in the exofacial leaflet) (Figure 7).

## Tilted peptides

There are several instances in which a cholesterol-binding site is functionally characterized in a TM segment without the help of the CRAC or CARC algorithms. This has recently been shown to be the case for α-synuclein, the neural protein associated with Parkinson disease, which can form oligomeric ion channels in the plasma membrane of neurons (Fantini and Yahi, 2010, 2011). This protein contains a CRAC domain which has been shown to bind cholesterol with low affinity. However, the domain is located outside the membrane-spanning regions of the proteins that supposedly interact with cholesterol and display neither CRAC nor CARC motifs (Fantini et al., 2011). Nevertheless, a second cholesterol-binding domain has been successfully characterized in α-synuclein, corresponding to a tilted peptide known to be toxic for cultured neurons, namely the segment 67-GGAVVTGVTAVA-78 (Fantini et al., 2011). What is intriguing about this peptide is that it does not contain the basic and aromatic residues that are mandatory in both the CRAC and CARC algorithms. Indeed, the definition of tilted peptides is functional, not sequence-based. Tilted peptides are short helical protein fragments that are able to disturb the organization of the molecular system into which they insert. They are characterized by an asymmetric distribution of their hydrophobic residues, which induces a tilted orientation (around 45°) toward the membrane plane (Lins et al., 2008). Because they induce a significant distortion of the membrane structure, tilted peptides are involved in the fusion process triggered by viral glycoproteins (Charloteaux et al., 2009). This is the case with the tilted peptide of α-synuclein, which binds cholesterol with a tilt angle of 46° (Crowet et al., 2007). Despite the lack of aromatic residues, the contribution of apolar aliphatic residues accounts for a total energy of interaction of −53 kJ.mol^−1^, which is comparable to the values obtained for both CRAC and CARC domains. This further illustrates how efficient van der Waals interactions between branched apolar residues and the β face of cholesterol can be. Similarly, a linear motif (22-EDVGSNKGAIIGLM-35) including a part of the tilted domain of Alzheimer's β-amyloid peptide has been identified as a high affinity binding site for cholesterol (Di Scala et al., 2013).

**Figure 8:**
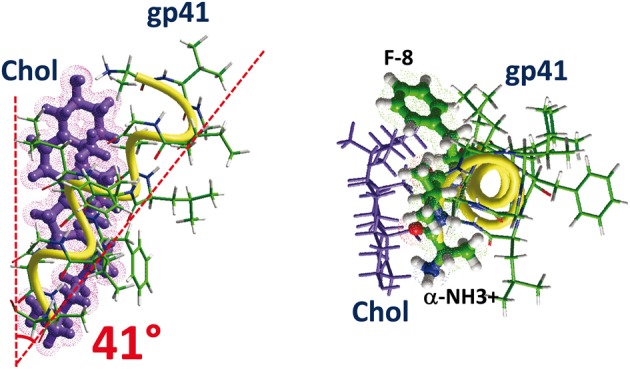
**How cholesterol interacts with a tilted peptide.** Docking of cholesterol with the N-terminal part of HIV-1 gp41, i.e., the fusion peptide. The apolar part of cholesterol interacts with the aromatic ring of F-8 through CH-π (but not stacking) interactions. The OH group of cholesterol is close to the α-NH3^+^ group of the peptide (N-terminus ending). The angle between the helix axis of the tilted fragment of the fusogenic tilted peptide of gp41 is 41°. The calculated energy of interaction is −48.5 kJ.mol^−1^.

An example of the interaction between a fusogenic tilted peptide and cholesterol is shown in Figure 8. This is the tilted peptide of the transmembrane glycoprotein gp41 of HIV-1. The tilted orientation of the peptide with respect to cholesterol is clearly visible in the model, and the tilt angle of 41° is close to the experimental value (Charloteaux et al., 2006). The association of the tilted peptide with cholesterol is mediated by a series of van der Waals interactions for a total energy of interaction of −48.5 kJ.mol^−1^. Tilted peptides have been detected in various proteins otherwise known to require cholesterol for their membrane insertion process, including amyloidogenic and viral fusion proteins (Fantini et al., 2011). This suggests that tilted peptides have evolved in such a way as to acquire the cholesterol-binding properties that facilitate their biological functions.

## Other cholesterol-binding motifs

An important issue to resolve is whether cholesterol can interact with TM domains of protein lacking a CRAC, a CARC or a tilted domain. If we consider any possible interaction between a membrane protein and its surrounding lipids, including cholesterol, then the answer might be yes. In this respect, two distinct types of lipid-protein interactions in the membrane environment should be considered. Lipids in the first boundary shell, or annulus, are referred to as annular or belt lipids (see review in Marsh and Pali, 2012). Such annular lipids interact with the membrane-embedded surface of the protein (Lee, 2011). In addition, there is a second category of lipids that are buried to a greater or lesser extent in the protein. Such lipids are found bound between TM domains and are referred to as non-annular lipids (Lee, 2011; Marsh and Pali, 2012). Because they are bound to the protein surface, annular lipids can be exchanged for other membrane lipids. This is usually not the case for non-annular lipids that are less or not accessible at all to surrounding membrane lipids. Indeed, non-annular binding sites may involve several TM domains that form a 3D binding site for the lipid. For instance, several potential cholesterol-binding residues located in distinct TM domains of the 5-HT1A serotonin receptor were identified by comparison with the conserved cholesterol-consensus motifs in the β2-adrenergic receptor—as predicted from its crystal structure (Paila et al., 2009). Interestingly, the evolutionary conserved residues of this non-annular binding site are typically those found in CRAC and CARC domains, i.e., a combination of aromatic (Y-73 in TM2, W-161 in TM4), basic (R-151 in TM4) and aliphatic (I-157 in TM4) residues. Thus, the molecular mechanisms of cholesterol binding described for the CRAC and the CARC domains should probably also apply for this 3D binding site.

Overall, the interaction between membrane proteins (or peptides) and annular/non-annular lipids has been studied by three main approaches: ^2^H nuclear magnetic resonance (NMR), spin-label electron paramagnetic resonance (ESR) and X-ray crystallography (see reviews in Marsh, 2008, 2010). This has enabled determination of the stoichiometry of the lipid–protein interaction, the selectivity of the protein for different lipids and the amino acid residues physically involved in lipid binding. X-ray crystallography is of particular value since it can give a precise description of lipid-protein contacts at the atomic scale.

Unfortunately, very few 3D structures of protein-cholesterol complexes are available in the literature. In 2008, Hanson et al. published the 2.8Å resolution crystal structure of a thermally-stabilized human β_2_-adrenergic receptor bound to cholesterol. The cholesterol binding site of this receptor was defined as a 3D motif involving the 1st, 2nd, 3rd, and 4th TM domains (referred to as TM1, TM2, TM3, and TM4). Among these domains, TM2 and TM4 appeared particularly critical for cholesterol binding. Interestingly, TM4 contains an aromatic tryptophan residue (W-158) that is highly conserved among human G-protein coupled receptors. The aromatic side chain of W-158 stacks onto cholesterol through a CH-Pi interaction with ring D of the sterol. The binding site also involves two other amino acid residues of TM4 (R-151 and L-163) and a second aromatic residue located in TM2 (Tyr-70).

As noted by Hanson et al. (2008), the amino acid sequence of TM4 contains the combination of basic (R), aromatic (W) and aliphatic (L/V) residues found in CRAC domains **(**151-**R**VIILMV**W**IVSG**L**TSFLPIQMHWY-174). However, this sequence does not fulfill the criteria of the CRAC domain. Moreover, it is even closer to a CARC domain (with the typical R—W—L motif), except that there are 6 residues between R-151 and W-158, which exceeds by one unit the maximal number of 5 residues allowed by the CARC algorithm. Finally, the unique spatial distribution of amino acid residues that are important for cholesterol interaction was used to define a consensus 3D binding motif among human G-protein-coupled receptors as follows: [4.39–4.43(R,K)]—[4.50(W,Y)]—[4.46(I,V,L)]—[2.41(F,Y)], according to the Ballesteros–Weinstein numbering scheme (Ballesteros and Weinstein, 1995).

Besides the human β_2_-adrenergic receptor, very few membrane proteins have been co-crystallized with cholesterol. NMR studies have been recently performed on mixed cholesterol/phosphatidylcholine micelles containing C99, a proteolytic fragment of Alzheimer's amyloid precursor protein (APP) (Barrett et al., 2012). Although this protein does not contain a CRAC or a CARC domain, its unique TM domain displays a cholesterol binding site consisting of a series of scattered Gly residues (GXXXG motifs) that confer an unusual high flexibility on the TM domain. These glycine residues are involved in cholesterol binding, which is chiefly mediated by van der Waals interactions between the apolar moiety of cholesterol and apolar residues within the motif (i.e., the aliphatic Ala, Ile, Val, and the aromatic Phe).

## Conclusions and perspectives

At first glance, this review of cholesterol-binding sites encased in TM domains of proteins may appear rather complex. Two consensus domains, CRAC and CARC, have been characterized. As a cholesterol-binding domain present in a TM domain, CARC appears more consistent than CRAC in predicting cholesterol-recognition motifs in integral membrane proteins, especially for predicting cholesterol-binding sites located in the exofacial leaflet of the plasma membrane. The CRAC algorithm is generally relevant (Sengupta and Chattopadhyay, 2012), but it can occasionally mis-predict unrealistic cholesterol-binding domains outside TM domains (Figure 4B). Fusogenic tilted peptides lacking a CARC or a CRAC domain can interact with cholesterol, although they do not contain the basic and aromatic amino acid residues that are mandatory for both the CARC and CRAC algorithms. Finally non-annular cholesterol-binding sites can be formed by the cooperation of several TM domains of G-protein coupled receptors, rendering the prediction of such binding domains particularly difficult.

Linear and 3D binding sites for cholesterol might have distinct biological functions. In bringing together several TM domains, cholesterol could exert a condensing effect on the whole protein, which, e.g., in the case of the 5-HT1 A receptor could help the protein to acquire some of its functional characteristics, including the delineation of the ligand-binding pocket (Paila et al., 2011). In contrast, cholesterol interacting with only one TM domain at the periphery of a receptor protein would be more suited to bringing together two distinct receptor macromolecules and triggering their dimerization, a key step in signal transduction cascades (Figure 3C).

This may explain why various types of cholesterol-binding motifs can be found in TM domains. These motifs may vary greatly in length, amino acid sequence, and can either be linear such as the CRAC (Li and Papadopoulos, 1998), CARC (Baier et al., 2011), and tilted peptides (Fantini et al., 2011) or three-dimensional, e.g., cholesterol consensus motifs (Hanson et al., 2008) and sterol sensing domains (Garmy et al., 2005). Nevertheless, common molecular mechanisms seem to control the interaction of transmembrane proteins with cholesterol. These mechanisms include the delineation of a polar area to accommodate the OH group of cholesterol at the membrane-water interface, and the establishment of numerous van der Waals interactions in the apolar zone of the membrane (Epand et al., 2006; Baier et al., 2011). These van der Waals interactions comprise London forces and CH-π interactions. London forces require an optimal geometric fit between cholesterol and the apolar side chains of the amino acid residues that belong to the motif. This explains why branched amino acid residues such as Ile, Val, and Leu are overrepresented in cholesterol-binding sites. Aromatic residues can stack onto the smooth α face of cholesterol or intercalate between the aliphatic spikes emerging from the rough β face of cholesterol. These simple biochemical rules probably apply to most if not all cholesterol-binding sites that are part of TM domains of proteins.

### Conflict of interest statement

The authors declare that the research was conducted in the absence of any commercial or financial relationships that could be construed as a potential conflict of interest.

## References

[B1] Anderson R. G. Jacobson K. (2002). A role for lipid shells in targeting proteins to caveolae, rafts, and other lipid domains. Science 296, 1821–1825 10.1126/science.1068886 12052946

[B2] Baier C. J. Fantini J. Barrantes F. J. (2011). Disclosure of cholesterol recognition motifs in transmembrane domains of the human nicoticin acetylcholine receptor. Sci. Reports 1:69 10.1038/srep00069 22355588 PMC3216556

[B3] Ballesteros J. Weinstein H. (1995). Integrated methods for the construction of three-dimensional models and computational probing of structure-function relations in G protein-coupled receptors. Methods Neurosci. 25, 366–428

[B4] Barrantes F. J. Borroni V. Vallés S. (2010). Neuronal nicotinic acetylcholine receptor-cholesterol crosstalk in Alzheimer's disease. FEBS Lett. 584, 1856–1863 10.1016/j.febslet.2009.11.036 19914249

[B5] Barrett P. J. Song Y. Van Horn W. D. Hustedt E. J. Schafer J. M. Hadziselimovic A. (2012). The amyloid precursor protein has a flexible transmembrane domain and binds cholesterol. Science 336, 1168–1171 10.1126/science.1219988 22654059 PMC3528355

[B6] Charloteaux B. Lorin A. Brasseur R. Lins L. (2009). The “Tilted Peptide Theory” links membrane insertion properties and fusogenicity of viral fusion peptides. Protein Pept. Lett. 16, 718–725 19601900 10.2174/092986609788681724

[B7] Charloteaux B. Lorin A. Crowet J. M. Stroobant V. Lins L. Thomas A. (2006). The N-terminal 12 residue long peptide of HIV gp41 is the minimal peptide sufficient to induce significant T-cell-like membrane destabilization *in vitro*. J. Mol. Biol. 359, 597–609 10.1016/j.jmb.2006.04.018 16677669

[B8] Crowet J. M. Lins L. Dupiereux I. Elmoualija B. Lorin A. Charloteaux B. (2007). Tilted properties of the 67-78 fragment of alpha-synuclein are responsible for membrane destabilization and neurotoxicity. Proteins 68, 936–947 10.1002/prot.21483 17554782

[B9] Di Scala C. Yahi N. Lelièvre C. Garmy N. Chahinian H. Fantini J. (2013). Biochemical identification of a linear cholesterol-binding domain within Alzheimer's β amyloid peptide. ACS Chem. Neurosci, (in press). 10.1021/cn300203a PMC3605813 23509984

[B10] Epand R. F. Thomas A. Brasseur R. Vishwanathan S. A. Hunter E. Epand R. M. (2006). Juxtamembrane protein segments that contribute to recruitment of cholesterol into domains. Biochemistry 45, 6105–6114 10.1021/bi060245+ 16681383 PMC2515711

[B11] Epand R. M. (2006). Cholesterol and the interaction of proteins with membrane domains. Prog. Lipid Res. 45, 279–294 10.1016/j.plipres.2006.02.001 16574236

[B12] Epand R. M. Thomas A. Brasseur R. Epand R. F. (2010). Cholesterol interaction with proteins that partition into membrane domains: an overview. Subcell. Biochem. 51, 253–278 10.1007/978-90-481-8622-8_9 20213547

[B13] Fantini J. Barrantes F. J. (2009). Sphingolipid/cholesterol regulation of neurotransmitter receptor conformation and function. Biochim. Biophys. Acta 1788, 2345–2361 10.1016/j.bbamem.2009.08.016 19733149

[B14] Fantini J. Carlus D. Yahi N. (2011). The fusogenic tilted peptide (67-78) of α-synuclein is a cholesterol binding domain. Biochim. Biophys. Acta 1808, 2343–2351 10.1016/j.bbamem.2011.06.017 21756873

[B15] Fantini J. Garmy N. Mahfoud R. Yahi N. (2002). Lipid rafts: structure, function and role in HIV, Alzheimer's and prion diseases. Expert Rev. Mol. Med. 4, 1–22 10.1017/S1462399402005392 14987385

[B16] Fantini J. Yahi N. (2010). Molecular insights into amyloid regulation by membrane cholesterol and sphingolipids: common mechanisms in neurodegenerative diseases. Expert Rev. Mol. Med. 12:e27 10.1017/S1462399410001602 20807455 PMC2931503

[B17] Fantini J. Yahi N. (2011). Molecular basis for the glycosphingolipid-binding specificity of α-synuclein: key role of tyrosine 39 in membrane insertion. J. Mol. Biol. 408, 654–669 10.1016/j.jmb.2011.03.009 21396938

[B18] Garmy N. Taïeb N. Yahi N. Fantini J. (2005). Interaction of cholesterol with sphingosine: physicochemical characterization and impact on intestinal absorption. J. Lipid Res. 46, 36–45 10.1194/jlr.M400199-JLR200 15520452

[B19] Gimpl G. (2010). Cholesterol-protein interaction: methods and cholesterol reporter molecules. Subcell. Biochem. 5, 1–45 10.1007/978-90-481-8622-8_1 20213539

[B20] Hanson M. A. Cherezov V. Griffith M. T. Roth C. B. Jaakola V. P. Chien E. Y. (2008). A specific cholesterol binding site is established by the 2.8 Å structure of the human beta2-adrenergic receptor. Structure 16, 897–905 10.1016/j.str.2008.05.001 18547522 PMC2601552

[B21] Harris J. R. Palmer M. (2010). Cholesterol specificity of some heptameric beta-barrel pore-forming bacterial toxins: structural and functional aspects. Subcell. Biochem. 51, 579–596 10.1007/978-90-481-8622-8_21 20213559

[B22] Harris J. S. Epps D. E. Davio S. R. Kézdy F. J. (1995). Evidence for transbilayer, tail-to-tail cholesterol dimers in dipalmitoylglycerophosphocholine liposomes. Biochemistry 34, 3851–3857 7893682 10.1021/bi00011a043

[B23] Ikonen E. (2008). Cellular cholesterol trafficking and compartmentalization. Nat. Rev. Mol. Cell Biol. 9, 125–138 10.1038/nrm2336 18216769

[B24] Jafurulla M. Tiwari S. Chattopadhyay A. (2011). Identification of cholesterol recognition amino acid consensus (CRAC) motif in G-protein coupled receptors. Biochem. Biophys. Res. Commun. 404, 569–573 10.1016/j.bbrc.2010.12.031 21146498

[B25] Jamin N. Neumann J. M. Ostuni M. A. Vu T. K. Yao Z. X. Murail S. (2005). Characterization of the cholesterol recognition amino acid consensus sequence of the peripheral-type benzodiazepine receptor. Mol. Endocrinol. 19, 588–594 10.1210/me.2004-0308 15528269

[B26] Lee A. G. (2003). Lipid-protein interactions in biological membranes: a structural perspective. Biochim. Biophys. Acta 1612, 1–40 10.1016/S0005-2736(03)00056-7 12729927

[B27] Lee A. G. (2011). Lipid-protein interactions. Biochem. Soc. Trans. 39, 761–766 10.1042/BST0390761 21599646

[B28] Li H. Papadopoulos V. (1998). Peripheral-type benzodiazepine receptor function in cholesterol transport. Identification of a putative cholesterol recognition/interaction amino acid sequence and consensus pattern. Endocrinology 139, 4991–4997 10.1210/en.139.12.4991 9832438

[B29] Lins L. Decaffmeyer M. Thomas A. Brasseur R. (2008). Relationships between the orientation and the structural properties of peptides and their membrane interactions. Biochim. Biophys. Acta 1778, 1537–1544 10.1016/j.bbamem.2008.04.006 18501700

[B30] Marsh D. (2008). Protein modulation of lipids, and vice-versa, in membranes. Biochim. Biophys. Acta 1778, 1545–1575 10.1016/j.bbamem.2008.01.015 18294954

[B31] Marsh D. (2010). Electron spin resonance in membrane research: protein–lipid interactions from challenging beginnings to state of the art. Eur. Biophys. J. 39, 513–525 10.1007/s00249-009-0512-3 19669751 PMC2841276

[B32] Marsh D. Pali T. (2012). Orientation and conformation of lipids in crystals of transmembrane proteins. Eur. Biophys. J. [Epub ahead of print]. 10.1007/s00249-012-0816-6 22644500

[B33] Mattjus P. Slotte J. P. (1996). Does cholesterol discriminate between sphingomyelin and phosphatidylcholine in mixed monolayers containing both phospholipids? Chem. Phys. Lipids 81, 69–80 10.1016/0009-3084(96)02535-2 9450320

[B34] Mesmin B. Maxfield F. R. (2009). Intracellular sterol dynamics. Biochim. Biophys. Acta 1791, 636–645 10.1016/j.bbalip.2009.03.002 19286471 PMC2696574

[B35] Mondal M. Mesmin B. Mukherjee S. Maxfield F. R. (2009). Sterols are mainly in the cytoplasmic leaflet of the plasma membrane and the endocytic recycling compartment in CHO cells. Mol. Biol. Cell 20, 581–588 10.1091/mbc.E08-07-0785 19019985 PMC2626560

[B36] Mukherjee S. Chattopadhyay A. (1996). Membrane organization at low cholesterol concentrations: a study using 7-nitrobenz-2-oxa-1, 3-diazol-4-yl-labeled cholesterol. Biochemistry 35, 1311–1322 10.1021/bi951953q 8573588

[B37] Nishio M. Umezawa Y. Hirota M. Takeuchi Y. (1995). The CH/π interaction: significance in molecular recognition. Tetrahedron 51, 8665–8701

[B38] Oddi S. Dainese E. Fezza F. Lanuti M. Barcaroli D. De Laurenzi V. (2011). Functional characterization of putative cholesterol binding sequence (CRAC) in human type-1 cannabinoid receptor. J. Neurochem. 116, 858–865 10.1111/j.1471-4159.2010.07041.x 21214565

[B39] Paila Y. D. Chattopadhyay A. (2010). Membrane cholesterol in the function and organization of G-protein coupled receptors. Subcell. Biochem. 5, 439–466 10.1007/978-90-481-8622-8_16 20213554

[B40] Paila Y. D. Tiwari S. Chattopadhyay A. (2009). Are specific nonannular cholesterol binding sites present in G-protein coupled receptors? Biochim. Biophys. Acta 1788, 295–302 10.1016/j.bbamem.2008.11.020 19111523

[B41] Paila Y. D. Tiwari S. Sengupta D. Chattopadhyay A. (2011). Molecular modeling of the human serotonin (1A) receptor: role of membrane cholesterol in ligand binding of the receptor. Mol. Biosyst. 7, 224–234 10.1039/c0mb00148a 20967314

[B42] Palmer M. (2004). Cholesterol and the activity of bacterial toxins. FEMS Microbiol. Lett. 238, 281–289 10.1016/j.femsle.2004.07.059 15358412

[B43] Radhakrishnan A. Anderson T. G. McConnell H. M. (2000). Condensed complexes, rafts, and the chemical activity of cholesterol in membranes. Proc. Natl. Acad. Sci. U.S.A. 97, 12422–12427 10.1073/pnas.220418097 11050164 PMC18778

[B44] Rose I. A. Hanson K. R. Wilkinson K. D. Wimmer M. J. (1980). A suggestion for naming faces of ring compounds. Proc. Natl. Acad. Sci. U.S.A. 77, 2439–2441 16592816 10.1073/pnas.77.5.2439 PMC349414

[B45] Rukmini R. Rawat S. S. Biswas S. C. Chattopadhyay A. (2001). Cholesterol organization in membranes at low concentrations: effects of curvature stress and membrane thickness. Biophys. J. 81, 2122–2134 10.1016/S0006-3495(01)75860-2 11566783 PMC1301684

[B46] Segrest J. P. De Loof H. Dohlman J. G. Brouillette C. G. Anantharamaiah G. M. (1990). Amphipathic helix motif: classes and properties. Proteins 8, 103–117 10.1002/prot.340080202 2235991

[B47] Sengupta D. Chattopadhyay A. (2012). Identification of cholesterol binding sites in the serotonin1A receptor. J. Phys. Chem. B 116, 12991–12996 10.1021/jp309888u 23067252

[B48] Simons K. Ikonen E. (1997). Functional rafts in cell membranes. Nature 387, 569–572 10.1038/42408 9177342

[B49] Strandberg E. Killian J. A. (2003). Snorkeling of lysine side chains in transmembrane helices: how easy can it get? FEBS Lett. 544, 69–73 10.1016/S0014-5793(03)00475-7 12782292

[B50] Thathiah A. De Strooper B. (2011). The role of G protein-coupled receptors in the pathology of Alzheimer's disease. Nat. Rev. Neurosci. 12, 73–87 10.1038/nrn2977 21248787

